# Prevalence and Burden of Self-Reported Health Problems in Junior Male Elite Ice Hockey Players: A 44-Week Prospective Cohort Study

**DOI:** 10.1177/03635465211032979

**Published:** 2021-08-16

**Authors:** Anine Nordstrøm, Roald Bahr, Ben Clarsen, Ove Talsnes

**Affiliations:** † Oslo Sports Trauma Research Center, Department of Sports Medicine, Norwegian School of Sports Sciences, Oslo, Norway; ‡ Sykehuset Innlandet HF, Innlandet Hospital Trust, Elverum, Norway; § Center for Disease Burden, Norwegian Institute of Public Health, Bergen, Norway; ‖ University of Oslo, Oslo University Hospital, Oslo, Norway; Investigation performed at Oslo Sports Trauma Research Center and Innlandet Hospital Trust, Oslo, Norway

**Keywords:** overuse injuries, ice hockey, epidemiology, injury prevention, junior injuries, adolescent injuries

## Abstract

**Background:**

Little is known about the burden of overuse injuries and illnesses in junior elite ice hockey.

**Purpose:**

To describe the prevalence and burden of all health problems in junior male elite ice hockey players in Norway during 1 school year.

**Study Design:**

Descriptive epidemiological study.

**Methods:**

A total of 206 junior male ice hockey players (mean age, 17 years; range, 15-20 years) attending specialized sports academy high schools in Norway reported all health problems, acute injuries, overuse injuries, and illnesses, weekly during the 2018-2019 school year (44 weeks). The players self-reported injuries and illnesses using the Oslo Sports Trauma Research Center Questionnaire on Health Problems.

**Results:**

Of the players included, 25% (95% CI, 23%-27%) reported at least 1 health problem at any given time, while 16% (95% CI, 14%-17%) experienced health problems with a substantial negative effect on training and performance. Of the total burden of health problems, acute injuries accounted for 44%, overuse injuries 31%, and illnesses 25%. For acute injuries, the greatest burden was caused by injuries to the ankle, knee, and hand, whereas for overuse injuries the most burdensome location was the hip/groin and knee.

**Conclusion:**

This study documented that while acute injuries did represent the greatest problem among junior elite ice hockey players, overuse injuries, especially to the knee and hip/groin, also had a substantial effect.

Ice hockey is a contact sport where players are at a high risk of injury. Players frequently collide with each other, boards, and goals, and get hit by sticks and pucks. The risk of injury in ice hockey increases with age.^22,25^ In junior, intercollegiate, and high school ice hockey, overall injury rates have been reported to be 9.4 per 1000 player-hours,^23^ 10.22 per 1000 athlete-exposures (AE),^17^ and between 34.4 and 96.1 per 1000 player-game hours, respectively.^22^ This variation in injury rates can likely be explained by methodological differences between studies. Nevertheless, injury risk is substantially higher during games (14.73 per 1000 AE,^17^ and 30.3-49.7^22^ and 96.1^23^ per 1000 player-game hours) than during training (2.52 per 1000 AE,^17^ and 3.9 per 1000 player-practice hours^23^). The head,^23,25^ shoulder,^17,23,25^ and knee^17,22^ are the most commonly injured body regions.

The majority of previous epidemiological studies in junior ice hockey have used traditional sports injury surveillance methods and a time loss or medical attention injury definition.^15,22,23^ Although this may be sufficient to capture traumatic injuries, injury definitions based only on time loss or medical attention most likely underestimate the total effect of overuse injuries.^3,10^ This is because overuse injuries often have a gradual onset and players commonly continue to participate despite persistent injury-associated symptoms and limitations.^3,7,10^ Listola^16^ found that 29.6% of all injuries in their study on junior players were overuse injuries, but injury was not clearly defined. Previous studies on elite ice hockey players have suggested that overuse injuries represent a relevant problem.^6,15^ A previous study on professional ice hockey players in the Norwegian premier professional league (GET League) did capture a greater burden from overuse injuries than traditional injury registration, but acute injuries still represented the main problem.^20^ In some other sports, the burden of overuse injuries and illnesses has been shown to be comparable with that of acute injuries.^1,8,10,11^ The true burden, defined as the cross-product of severity and incidence, of overuse injuries in junior ice hockey is unknown (as is the burden of illness).

In this study, our aim was to describe the prevalence and burden of all health problems affecting junior elite ice hockey players in specialized sports academy high schools in Norway. This information can inform the development of effective injury and illness prevention protocols.

## Methods

### Study Design and Participants

This was a prospective cohort study among junior elite ice hockey players in 5 schools and 1 club during the 2018-2019 school year. The methods used are the same as those we recently applied in a similar study of the Norwegian professional ice hockey league.^20^ A total of 206 male players (mean age, 17 years; range, 15-20 years) registered all health problems once a week using a smartphone or tablet. The study was approved by the Norwegian Centre for Research Data (ref. 59423 LH/LR) and reviewed by the Norwegian Data Inspectorate (ref. 17/00803-6/CGN), and all players provided written informed consent to participate in the study. For those aged under 18 years, written consent was signed by their parents. All participants approved access to their data for their school staff and physician.

### Recruitment and Inclusion Criteria

We included 6 private specialized sports academy high schools that offer elite sports programs to students who want to combine sports on a high level with a college-entry academic program. The principal investigator (A.N.) contacted the schools, their management, and their coaches by email and phone in January 2018 and held meetings with all schools providing information about the study during the winter and spring of 2018. The schools had limited medical support; one of the schools had a physician, the rest had dedicated school staff who contacted medical personnel when needed. We informed the players about the study during a meeting at their school at the start of the school year. We wanted to include 6 schools. However, 1 school preferred that the study would be conducted through their local affiliated club. We agreed to enroll 2 teams (under 18 years and under 21 years) in the study. In the 5 schools and 1 club included, 3 players declined the invitation to participate in the study, 2 players did not respond in the system after they agreed to participate, and 4 players stopped reporting after 18 weeks. A total of 23 players dropped out throughout the year because of change of school, change of team, or unknown reasons. One school had 1 female player whom we excluded from the analyses. The final study sample consisted of 206 players.

### Injury and Illness Data Collection

Surveillance was conducted using an online platform designed to collect injury and illness data from athletes using the Oslo Sports Trauma Research Center Questionnaire on Health Problems (OSTRC-H2)^9-11^ (AthleteMonitoring.com; Fitstats Inc). All players received SMS messages and/or emails weekly with a direct link to the AthleteMonitoring website. The OSTRC-H2 was distributed automatically once a week (every Monday) from August 6, 2018, until June 10, 2019 (44 weeks). One school (n = 35) started registration August 6, 1 school (n = 33) August 13, 1 school and 1 club (n = 80) September 3, and 2 schools (n = 58) September 10. If players failed to complete the questionnaire, the system sent an automated reminder every day until a response was received. Additionally, the principal investigator (A.N.) sent SMS reminders to nonresponders after 3 and 5 days. The school physician and the dedicated staff members at the other schools and club involved in the project could access their players’ health information on a web-based dashboard and encouraged players to respond. To encourage participation, the principal investigator visited 3 of the 5 participating schools during December 2018 and January 2019 and maintained regular contact with all players and responsible staff members throughout the registration period.

### Oslo Sports Trauma Research Center Questionnaire on Health Problems

The OSTRC-H2 consists of 4 graded questions about the athlete’s participation in sports, modification in training or competition, performance, and symptoms of health problems during the past 7 days.^9-11^ The response to each of the 4 questions was allocated a numeric value between 0 and 25, where 0 represented no problems and 25 the maximum level for each question. Each question had 4 response options, and each response was scored 0-8-17-25. For each health problem, the responses in numeric values were summarized to calculate a severity score from 0 to 100, minimum 0 (full participation without health problems) and maximum 100 (no participation at all). If the athlete answered “no” on the first questions (full participation without problems), a total severity score of 0 was assigned and the questionnaire was completed for that week. If athletes reported a health problem, they were asked about modification in training or competition, performance, and symptoms and to define whether the health problem was an injury or an illness. In the case of an injury, they were asked to classify whether it was an acute injury (associated with a specific, clearly identifiable traumatic event) or an overuse injury (no specific identifiable event responsible for the occurrence) and register the affected anatomic area. The players could not specify if their injuries were a result of hockey participation. In the case of a reported illness, they were asked to report their main symptoms (by choosing from multiple predefined symptoms). For all types of health problems, athletes were asked to register the number of days of complete time loss from training and competition (total inability to train or compete) and whether the health problem had been reported previously. They were asked to register all health problems, and in cases of multiple problems the same week, the questionnaire repeated itself. Custom-made questions about the number of training sessions and games played, as well as information about sleep in the previous 7 days, were added to the questionnaire in November 2018.

### Definition and Classification of Health Problems

We used an “all complaints” definition, recording all health problems irrespective of the need for medical attention or the consequences on sports participation.^12,19,24^ Our definitions of health problem, acute injury, overuse injury, and illness were consistent with the International Olympic Committee consensus statement.^4^ The definition of an acute injury was an injury caused by a single, clearly identifiable energy transfer (eg, a fall or collision). The definition of an overuse injury was an injury caused by multiple accumulative bouts of energy transfer without a single, clearly identifiable event responsible for the injury. Health problems were defined as “substantial problems” if they caused moderate or severe modifications to training, moderate or severe reductions in performance, or a complete inability to participate in ice hockey.^9-11^ Players with health problems who selected option 4 in question 1, or option 3 or 4 in either question 2 or 3 in the questionnaire, were considered to have a “substantial problem.” Burden is a collective measure of the overall effect of a health problem.^4^ We defined it in several ways: the sum of severity scores over the duration of the study, the total number of time-loss days, and the cross-product of severity and incidence for all weeks in the questionnaire.^4,5^

### Prevalence Calculations

Each week, we calculated the following prevalence measures using the methods described by Clarsen et al^10^: all health problems, substantial health problems, all injuries, substantial injuries, all illnesses, and substantial illnesses. We also calculated the average weekly prevalence with 95% CIs.

### Incidence and Relative Burden of Acute Injury, Overuse Injury, and Illness

After reviewing each athlete’s questionnaire responses for the entire season, we compiled a list of cases that included the following information: type of health problem, location (for injuries) or main system affected (for illnesses), number of weeks reported, cumulative time-loss days, and cumulative severity score. We also categorized the severity of each case based on its cumulative time loss as slight (0 days), mild (1-7 days), moderate (8-28 days), or severe (>28 days). The incidence of each type of health problem was expressed as the number of cases per player per year (52 weeks). Player availability was determined based on player response to question 1 of the questionnaire each week.

To reflect the relative burden of acute injuries, overuse injuries, and illnesses as a proportion of the total health burden, we summarized the severity scores for each health problem type and divided the result by the cumulative severity score for all health problems.^5^

We also created risk matrices based on the severity and incidence of injuries in the most affected anatomic regions.^5^ This was performed separately for acute and overuse injuries, as well as illnesses, using 2 measures of severity: (1) the average number of time-loss days per case and (2) the average severity score per case.

### Data Analysis

All data were extracted using Microsoft Excel software (Microsoft Office 365 ProPlus Version 2002). All analyses of data were done in Microsoft Excel software and Stata (Version 16.0). Data collected in the first week of the registration of all athletes were not included in the calculations, as per previous recommendations.^10^

## Results

### Response Rate to the Weekly Questionnaires

We distributed 7894 questionnaires and received 6743 responses (average weekly response rate, 85%; range, 71%-95%) during the 44-week study period. The response rate fell slightly from the first weeks (90% during weeks 1-10) to the last weeks (80% during weeks 35-44) of registration.

### Number, Incidence, and Severity of Health Problems

The players reported 230 acute injuries, 142 overuse injuries, and 246 illnesses in total. This translates to 1.8 new acute injuries, 1.1 new overuse injuries, and 1.9 new illnesses per athlete per year (Table 1). The average time loss was 35 days per athlete per year (95% CI, 34-35 days), 17 days for acute injuries (95% CI, 16-19 days), 9 days for overuse injuries (95% CI, 7-10 days), and 9 days for illnesses (95% CI, 7-11 days).

**Table 1:** Number of Cases, Incidence, Total Time Loss, and Cumulative Severity Score of Acute Injuries, Overuse Injuries, and Illnesses

	No. of Cases	Incidence, Cases/Athlete/Year (95% CI)	Total Time Loss (days)	Cumulative Severity Score
Acute injury	230	1.8 (1.6-2.0)	2221	47,966
Overuse injury	142	1.1 (0.9-1.3)	1114	33,700
Illness	246	1.9 (1.7-2.2)	1139	27,855
Total	618	4.8 (4.4-5.2)	4477*^a^*	109,620*^b^*

a One player registered 3 days of time loss without registering any injury or illness.

b One player registered a cumulative severity score of 99 without registering any injury or illness.

The most frequent acute injury locations were the head, shoulder, knee, and ankle, and the most frequent overuse injury locations were the pelvis, hip/groin, and knee. The number and severity of acute injuries, overuse injuries, and illnesses are summarized by region and organ system as Supplementary Material (see Appendix Table A1, available in the online version of this article).

### Prevalence of Health Problems

The maximum number of registered health problems per athlete per week was 3. The average weekly prevalence of health problems, the range, and 95% CIs are shown in Table 2. The prevalence of all health problems remained relatively stable during the competitive season but decreased slightly during the last weeks of the registration as the players only attended training and did not play matches.

**Table 2:** Average Weekly Prevalence of All Health Problems and Substantial Health Problems During the 44-Week Study Period

Prevalence	Mean %	95% CI	Range
All health problems	25	23-27	12-48
Injuries	20	18-22	9-35
Acute injuries	10	9-11	3-18
Overuse injuries	10	9-11	5-20
Illness	6	5-7	1-14
Substantial health problems	16	14-17	7-27
Injuries	12	11-13	6-22
Acute injuries	7	6-8	2-13
Overuse injuries	5	4-5	3-9
Illness	4	4-5	1-9

### Player Availability

On average, 81% (range, 71%-93%) of players were fully available, 13% (range, 5%-18%) had modified participation, and 6% (range, 0%-12%) were unavailable because of health problems.

### Burden of Health Problems

Calculating the relative burden using the total number of time-loss days as the basis for injury severity (Table 1), we found that acute injuries represented 50%, overuse injuries 25%, and illnesses 25% of the total burden of health problems.

Using cumulative severity scores as the basis for injury severity, we found that acute injuries represented 44% of the total burden of health problems, with overuse injuries and illnesses representing 31% and 25%, respectively.

**Figure 1.**
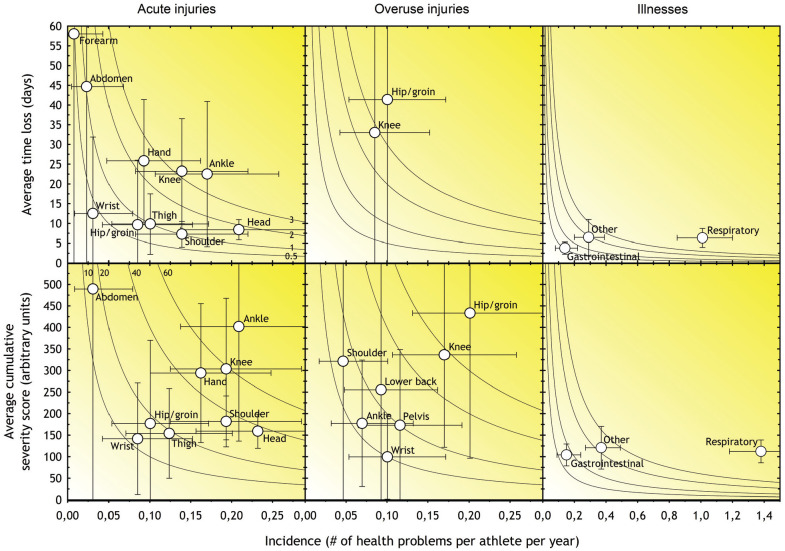
Risk matrices illustrating the relationship between severity (consequence) and incidence (likelihood) of the most commonly reported types of injuries in Norwegian junior elite ice hockey. For each injury in all matrices, incidence is shown as cases per athlete per year. The shading illustrates the relative importance of each of the injury types; the darker the color, the greater the injury burden and the greater the priority that should be given to prevention. The gray isobars show burdens of 0.5, 1, 2, and 3 days lost per year per athlete. The vertical and horizontal error bars represent 95% CIs. Regions with an average burden of <0.35 days for time loss and a cumulative severity score of <8 are not included in the figure.

The relationship between severity and incidence for the anatomic regions most affected by acute and overuse injury, respectively, is illustrated in Figure 1. The hand, knee, and ankle represented the greatest burden of acute injuries, while the hip/groin and knee represented the greatest burden of overuse injuries.

## Discussion

This paper presents the first prospective data on all injuries and illnesses, including those that did not lead to time loss, in junior elite ice hockey. The methods used allowed us to compare the relative burden of acute injuries, overuse injuries, and illnesses,^9-11^ providing a more comprehensive picture of the effect of health problems on sports participation and performance in this group of athletes. In our study, acute injuries represented 44%, overuse injuries 31%, and illnesses 25% of the total effect of health problems. Irrespective of how we calculated injury severity, acute injuries represented the greatest burden of health problems. These findings are consistent with our recent study on Norwegian professional ice hockey^20^ and in contrast to studies from other sports^10,11,18^ using the same methodology, which found that overuse injuries represented the greatest relative burden.

We found that, on average, 1 in 4 players reported a health problem and 1 in 6 a substantial health problem at any given time. This is less than a previous study on professional ice hockey players using the same methodology.^20^ This finding might be expected, as the risk of injury in ice hockey increases with age.^22,25^ To our knowledge, this is the first study within junior ice hockey using methodology designed to document different types of health problems. Hence, methodological differences make comparisons with other studies on junior ice hockey difficult. Compared with studies employing the same methodology in other sports, our findings were in line with observations by Pluim et al^21^ (average weekly prevalence of health problems, 21%; substantial health problems, 15%) and Clarsen et al^11^ (36% and 15%, respectively), but lower than those of Moseid et al^18^ (43% and 25%, respectively) and Gram et al^14^ (39% and 28%, respectively). However, the participant profile differs substantially between these studies: Clarsen et al^11^ monitored adult Olympic athletes from mixed sports; Pluim et al,^21^ 11- to 14-year-old elite tennis players; Moseid et al,^18^ a 15- to 16-year-old mixed group of youth athletes attending sports academy high schools, and Gram et al,^14^ female rhythmic gymnasts.

In our study, the average weekly prevalence of all acute injuries reported was 10% and the average weekly prevalence of substantial acute injuries was 7%. This is lower than the findings of Nordstrøm et al^20^ in professional ice hockey players, as may be expected because the rate of time-loss injuries in ice hockey has been shown to increase with age.^22,25^ Compared with studies using the same methodology in other sports, our findings are lower than the findings of Moseid et al,^18^ but greater than those of Clarsen et al^11^ and von Rosen et al^27^ in their study on adolescent elite orienteerers. Acute injuries to the hand, knee, and ankle caused the greatest burden. Knee and ankle injuries are largely consistent with previous epidemiological studies from junior ice hockey.^17,22,23,25^ However, as a consequence of different anatomic categorization methods, direct comparisons between studies are difficult.

The average weekly prevalence of overuse injuries in our study was 10%, about the same as acute injuries (10%). The prevalence of substantial overuse injuries was only 5%, meaning that few overuse injuries affected performance or participation. This rate is lower than previous findings in professional ice hockey^6,20^ and previous studies^2,10,11,14,18,27^ using the same methodology in other sports. The hip/groin and knee were the regions most commonly affected by overuse injuries, in accordance with previous findings in professional ice hockey.^6,20^ The burden of overuse injuries in our study is comparable with the main injured areas for acute injuries. The prevalence of overuse injuries should encourage ice hockey teams and organizations to emphasize the prevention of not only acute injuries but also overuse injuries. Junior ice hockey teams, coaches, and parents should target overuse injury prevention strategies focusing on the knee and hip/groin. In a small study of professional ice hockey players, strengthening the adductor muscle group appeared to be an effective method for preventing adductor strains,^26^ but large-scale intervention studies are not available from junior ice hockey yet.

Listola^16^ found overuse injuries accounted for 29% of injuries in a study on Finnish elite junior ice hockey players, which is lower than the rate in our study (38%). Listola studied fewer athletes (n = 53) over 1 season. As the author did not calculate the prevalence of injuries, it is difficult to compare those findings to ours. However, in our study, 206 players reported 142 cases of overuse injuries. Listola found that 19 players reported an overuse injury; 12 of these prevented full participation in games or practice sessions. It might be expected that several players had overuse injuries that did not affect participation during a season.

The average weekly prevalence of illnesses was 6%, with a prevalence of substantial illness of 4%. This is similar to a previous study on professional ice hockey players in Norway,^20^ but lower than those reported by Moseid et al,^18^ who studied Norwegian athletes of similar age and level but from a broad range of sports. In our study, illnesses represented about 25% of the total effect of health problems. However, our findings did not show that symptoms of illnesses had a great substantial effect on health, training, or performance. The average number of reported respiratory infections (1.39 per year; 95% CI, 1.20-1.61) (Figure 1) was lower compared with similar aged Norwegians in the general population (2.79; 95% CI, 1.87-4.10).^13^

Our data indicate that most acute injuries and illnesses in junior ice hockey are captured whether using a “time loss” or “all complaints” definition (Figure 1), suggesting that differences in injury definitions have little effect on the relative incidence, severity, and burden. In contrast, as the only overuse injuries that cause substantial time loss are knee and hip/groin injuries, using a different threshold to record overuse injuries will capture a greater amount of injuries and affect their relative incidence, severity, and burden.

### Methodological Considerations

One of the main strengths of the study is the high response rate. An additional strength is its full-season duration and relatively large sample size. We also used sensitive injury surveillance methods to capture all health problems.

This study also has several limitations. The data were collected from the players and the extent to which injuries were underreported could not be measured. The weekly reports by the athletes are subjective, and the reporting threshold may differ between players. In addition, as there may be systematic differences in reporting threshold between sports, comparisons with other studies should be interpreted with caution. Recall bias and underreporting of health problems could also affect the results; daily reports could reduce this bias but, on the other hand, challenge the compliance of the participants.

Recording of specific diagnoses has been achieved in previous studies using similar methods.^11^ Every time an athlete reported a health problem, medical teams/personnel registered a specific diagnosis. This requires close monitoring of reported cases by team medical staff, which was not possible in our study as only 1 of the schools had a physician. Health-related problems are expected in ice hockey. The wide definition used, based on “all health complaints,” leads to the registration of minor and transient problems (eg, muscle soreness or unspecific symptoms).^10^ This is a source of systematic bias, overestimating the prevalence of sports-related health problems. The “substantial health problem” definition (problems leading to reduced performance and/or participation) might be a more appropriate estimate of the effect of injuries and illnesses in ice hockey.

Our study only included male junior elite ice hockey players and may not be generalizable to other populations.

## Conclusion

At any given time, 1 in 4 male junior elite ice hockey players reported symptoms of injury or illness, and 1 in 6 of all players reported health problems that substantially affected their training or performance. This study documented that the total effect from overuse injuries, especially to the knee and hip/groin, was substantial; still, acute injuries did represent the main health problem. Future studies should evaluate potential risk factors for injuries and illnesses in ice hockey, like overall training and match load, physical fitness level, and total load.

## Supplemental Material

sj-pdf-1-ajs-10.1177_03635465211032979 – Supplemental material for Prevalence and Burden of Self-Reported Health Problems in Junior Male Elite Ice Hockey Players: A 44-Week Prospective Cohort Study Click here for additional data file. Supplemental material, sj-pdf-1-ajs-10.1177_03635465211032979 for Prevalence and Burden of Self-Reported Health Problems in Junior Male Elite Ice Hockey Players: A 44-Week Prospective Cohort Study by Anine Nordstrøm, Roald Bahr, Ben Clarsen and Ove Talsnes in The American Journal of Sports Medicine
