# Supplementary material for: Prevalence and Burden of Self-Reported Health Problems in Junior Male Elite Ice Hockey Players: A 44-Week Prospective Cohort Study
Source: Am J Sports Med. 2021 Aug 16;49(12):3379–85. doi: 10.1177/03635465211032979 (PMC8485253; doi:10.1177/03635465211032979)
Supplement: sj-pdf-1-ajs-10.1177_03635465211032979 – Supplemental material for Prevalence and Burden of Self-Reported Health Problems in Junior Male Elite Ice Hockey Players: A 44-Week Prospective Cohort Study [file sj-pdf-1-ajs-10.1177_03635465211032979.pdf]

Prevalence and burden of self-reported health problems in junior male elite ice hockey players. A 44-week prospective cohort study

Appendix Table A1. Number of cases and duration of time loss (days) of acute injuries, overuse injuries and illnesses by region and organ system.

	Cases (n)	Duration of time loss (n)				Total time loss (days)
		Slight (0 days)	Mild (1-7 days)	Moderate (8-28 days)	Severe (>28 days)	
**Acute injury**						
Head	30	3	12	14	1	235
Neck	6	4	1	1	0	22
Chest/ribs/upper back	11	5	5	1	0	34
Shoulder	25	7	12	6	0	131
Upper arm	1	0	1	0	0	3
Elbow	3	1	2	0	0	10
Forearm	3	2	0	0	1	58
Wrist	11	7	2	2	0	50
Hand	21	9	4	3	5	310
Abdomen	4	1	1	1	1	134
Lower back	9	3	5	1	0	27
Pelvis	7	2	4	1	0	27
Hip/groin	13	2	10	0	1	106
Thigh	16	3	8	3	2	128
Knee	25	7	9	3	6	418
Lower leg	2	1	1	0	0	1
Ankle	27	5	12	7	3	473
Foot	10	4	5	1	0	28
Non-specific	6	2	3	1	0	26
**Overuse injury**						
Neck	4	2	1	1	0	12
Chest/ribs/upper back	8	5	2	1	0	21
Shoulder	6	3	2	1	0	18
Elbow	2	2	0	0	0	0
Forearm	2	1	1	0	0	1
Wrist	13	9	4	0	0	13
Hand	7	5	2	0	0	7
Lower back	12	7	3	2	0	35
Pelvis	15	9	5	1	0	37
Hip/groin	26	13	3	5	5	538
Thigh	4	2	2	0	0	4
Knee	22	11	7	2	2	363
Lower leg	5	5	0	0	0	0
Ankle	9	5	2	1	1	45
Foot	7	3	3	1	0	20
**Illness**						
Respiratory	178	47	108	21	2	831
Gastrointestinal	20	2	16	2	0	68
Other illness	48	11	34	2	1	240
**Total**	618	210	292	85	31	4474
